# Summary report of the Standards, Options and Recommendations for malnutrition and nutritional assessment in patients with cancer (1999)

**DOI:** 10.1038/sj.bjc.6601089

**Published:** 2003-08-15

**Authors:** A Duguet, P Bachmann, Y Lallemand, M P Blanc-Vincent

**Affiliations:** 1 Centre Oscar Lambret, Lille, France; 2 Centre Léon Bérard, Lyon, France; 3 FNCLCC, Paris, France

**Keywords:** nutritional assessment, neoplasms-complications, practice guideline

In healthy subjects, nutritional ‘equilibrium’ is maintained by the oral intake of substrates providing energy, structural integrity and body regulation (vitamins, minerals, etc). Any reduction in intake of these elements, especially when combined with the metabolic abnormalities observed in patients with cancer, leads to an imbalance resulting in progressive malnutrition. This is generally a protein–calorie malnutrition, often accompanied by deficits in specific nutrients. The prevalence of malnutrition is dependent on the tumour site and its stage. Malnutrition is more prevalent in patients with cancer of the proximal gastrointestinal tract.

Malnutrition can be due to several factors:

- Reduced intake due to difficulties in eating, pain, mechanical obstruction and occasionally psychological disturbances.
- Metabolic changes induced by the tumour.
- Modification of energy expenditure.

## OBJECTIVES

The objective of this document is to define the ‘Standards’, ‘Options’ and ‘Recommendations’ (SORs) for nutritional assessment in adults with cancer and to describe the nutritional assessment tools for use in daily practice. Experimental and/or high-technology techniques, techniques for evaluating specific nutrient deficits and the investigation of organic or functional gut dysfunction are not covered here. The nutritional assessment of children is not covered in this document. Management of malnutrition and nutritional support techniques is covered in other specific SOR documents. This document will not aim to explain in detail the physiopathology of malnutrition in patients with cancer.

## METHODOLOGY

The general methodology used has already been described (Fervers *et al*, 2001). For this specific SOR, a working group of dietitians set up by the French National Federation of Cancer Centres (Féderation Nationale des Centres de Lutte Contre le Cancer; FNCLCC) reviewed the best available evidence on the nutritional assessment of patients with cancer.

A literature search was performed in *Medline*® up to March 1999 using the following terms: *nutrition assessment*, *nutritional status*, *neoplasms*. The references thus identified were completed by members of the working group from their personal reference lists. After selection and critical appraisal of this literature, the working group defined the ‘Standards’, ‘Options’ and ‘Recommendations’ for nutritional assessment of patients with cancer, based on a synthesis of the best available evidence.

When all the members of the working group agree, based on the best available evidence, that a procedure or intervention is beneficial, inappropriate or harmful, it is classified as a ‘Standard’, and when the majority agree, it is classified as an ‘Option’ (Table 1

**Table 1:** Definition of Standards, Options and Recommendations

Standards	Procedures or treatments that are considered to be of benefit, inappropriate or harmful by unanimous decision, based on the best available evidence
	
Options	Procedures or treatments that are considered to be of benefit, inappropriate or harmful by a majority, based on the best available evidence
	
Recommendations	Additional information to enable the available options to be ranked using explicit criteria (e.g. survival, toxicity) with an indication of the level of evidence

). In the SORs, there can be several ‘Options’ for a given clinical situation. ‘Recommendations’ provide additional information that enable the available options to be ranked using explicit criteria (e.g. survival, toxicity) with an indication of the level of evidence. These recommendations thus help clinicians to select an appropriate option. Thus, clinicians can make choices for the management of patients using this information and taking into consideration local circumstances, skills, equipment, resources and/or patient preferences. The adaptation of the SOR to the local situation is allowable if the reason for the choice is sufficiently transparent, and this is crucial for successful implementation. Inclusion of patients in clinical trials is an appropriate form of patient management in oncology and is recommended frequently within the SORs, particularly in situations where only weak evidence exists to support a procedure or an intervention.

The type of evidence underlying any ‘Standard’, ‘Option’ or ‘Recommendation’ is indicated using a classification developed by the FNCLCC based on previously published methods. The level of evidence depends not only on the type and quality of the studies reviewed, but also on the concordance of the results (Table 2

**Table 2:** Definition of level of evidence

*Level A*
There exists a high-standard meta-analysis several high-standard randomised clinical trials which give consistent results

*Level B*
There exist good quality evidence from randomised trials (B1) or prospective or retrospective studies (B2). The results are consistent when considered together

*Level C*
The methodology of the available studies is weak or their results are not consistent when considered together

*Level D*
Either the scientific data do not exist or there is only a series of cases

*Expert agreement*
The data do not exist- for the method concerned, but the experts are unanimous in their judgement

). When no clear scientific evidence exists, judgement is made according to the professional experience and consensus of the expert group (‘expert agreement’), and this is then validated by the peer-review process.

The working group drafted the full text version of the ‘Standards’, ‘Options’ and ‘Recommendation’. This version was then peer-reviewed by independent experts, whose comments were taken into consideration in the version that was validated in August 1999. The French summary version was prepared from the full text version and this is translated into English here. These documents will be updated when new scientific data become available or if there is a change in expert agreement.

This summary version is based on the full version (Duguet *et al*, 1999) and is also available on the FNCLCC web site: http://www.fnclcc.fr

## EFFECT OF ANTITUMOUR TREATMENT ON NUTRITION

Surgery induces metabolic changes that can be worsened by infectious complications thus increasing malnutrition. After major gastrointestinal surgery, the availability of nutrients can be reduced and oral food intake is lower. Hypercatabolism can occur in the perioperative phase. Increased mortality, local morbidity and infectious complications are observed in patients with severe malnutrition.

Chemotherapy can affect mucous membranes (mucositis) and can induce nausea, vomiting, anorexia and/or diarrhoea leading to reduced food intake. Hypercatabolism is sometimes seen in patients who undergo high-dose chemotherapy with peripheral blood stem cell support.

Radiotherapy has direct short-term and long-term effects on olfactory and secretory function, the digestive tract and the mucous membranes. Long-term bone, dental, olfactory, secretory and digestive sequelae can lead to reduced food intake and/or malabsorption of nutrients.

## CONSEQUENCES OF MALNUTRITION IN PATIENTS WITH CANCER

Malnutrition in patients with cancer is associated with a deterioration in quality of life and increased perioperative morbidity and mortality following major surgery. It can also lead to a lower response to treatment.

## NUTRITIONAL ASSESSMENT: CLINICAL AND ANTHROPOMETRIC DATA

The clinical and anthropometric assessment should include the measurement of height and weight, premorbid weight, amount and rate of weight loss, a calculation of body-mass index, collection of socioeconomic data, assessment of any digestive disorders, clinical examination and calculation of energy requirements (standard). The assessment can be completed by the measurement of skin fold thickness (option) or the mid-arm muscle circumference (option). Nutritional support is recommended when patients have lost more than 10% of their usual body weight over 6 months (recommendation). The World Health Organization (WHO) scale should be used to evaluate the toxicity of anticancer treatments on the digestive tract (recommendation, expert agreement).

## ASSESSMENT OF FOOD INTAKE

The assessment of food intake should include the calculation of the calorie–nitrogen ratio consumed (standard) using one of two techniques: dietary recall and dietary record (option).

## ASSESSMENT OF THE MODIFICATION OF FUNCTIONAL CAPACITIES

Functional capacity can be assessed using one of two validated tools: WHO performance status or the Karnofsky index (option). The same tool should be used throughout.

## CLINICAL AND NUTRITIONAL SCORES

Multidimensional clinical and nutritional assessment can be performed using scores based on one of the three validated scales: Detsky's subjective global assessment; the self-administered global assessment; and the mini nutritional assessment in elderly patients (option).

## LABORATORY DATA AND RISK FACTORS

Assays for albumin, prealbumin (or transthyretin), transferrin and retinol binding proteins can be used as biological markers for malnutrition in patients with no ongoing inflammatory condition (option). The predictive value of each individual parameter alone is not sufficient. Risk scores, combining several parameters, should be used (recommendation). Four tools are available for this: the prognostic inflammatory and nutritional index, the nutritional risk index, the prognostic nutritional risk and the aid for decision for nutritional support score (option). Functional and objective measurements can be made (bioelectric impedance analysis and calorimetry) for the follow-up and assessment of the degree of malnutrition and/or to assess metabolic requirements (option).

## MINIMUM ROUTINE NUTRITIONAL ASSESSMENT FIGURE 1)

**Figure 1:**
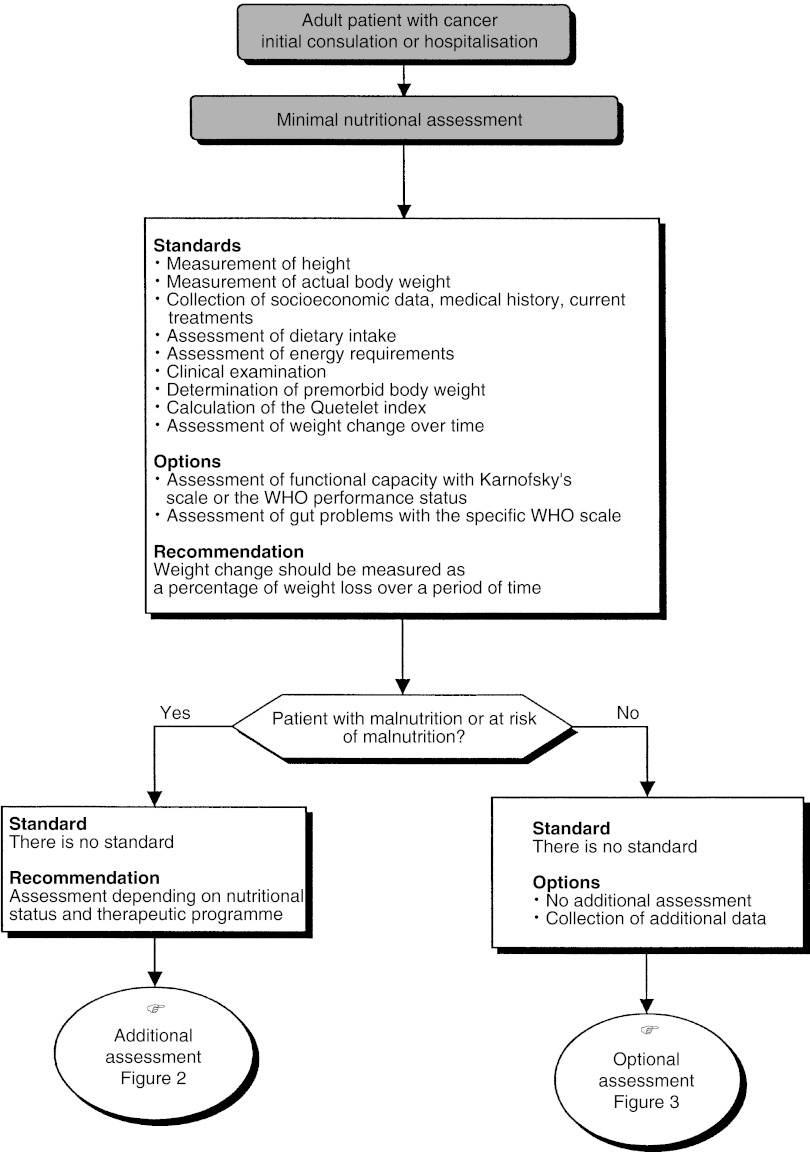
Minimal nutritional assessment.

The minimum routine nutritional assessment should include clinical history taking, measurement of the current height and weight, premorbid body weight, and the assessment of weight changes and dietary intake by a dietitian (standard). An assessment of functional capacity using a validated scale (WHO or Karnofsky) and an assessment of gastrointestinal disorders using the WHO scale can be undertaken (option). Weight change, over a given period, should be measured and presented as a percentage of weight loss compared with the premorbid body weight (recommendation).

## ADDITIONAL NUTRITIONAL ASSESSMENT (FIGURES 2 AND 3)

**Figure 2:**
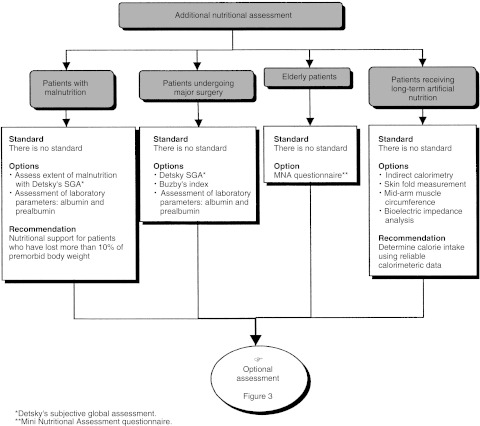
Additional nutritional assessment.

**Figure 3:**
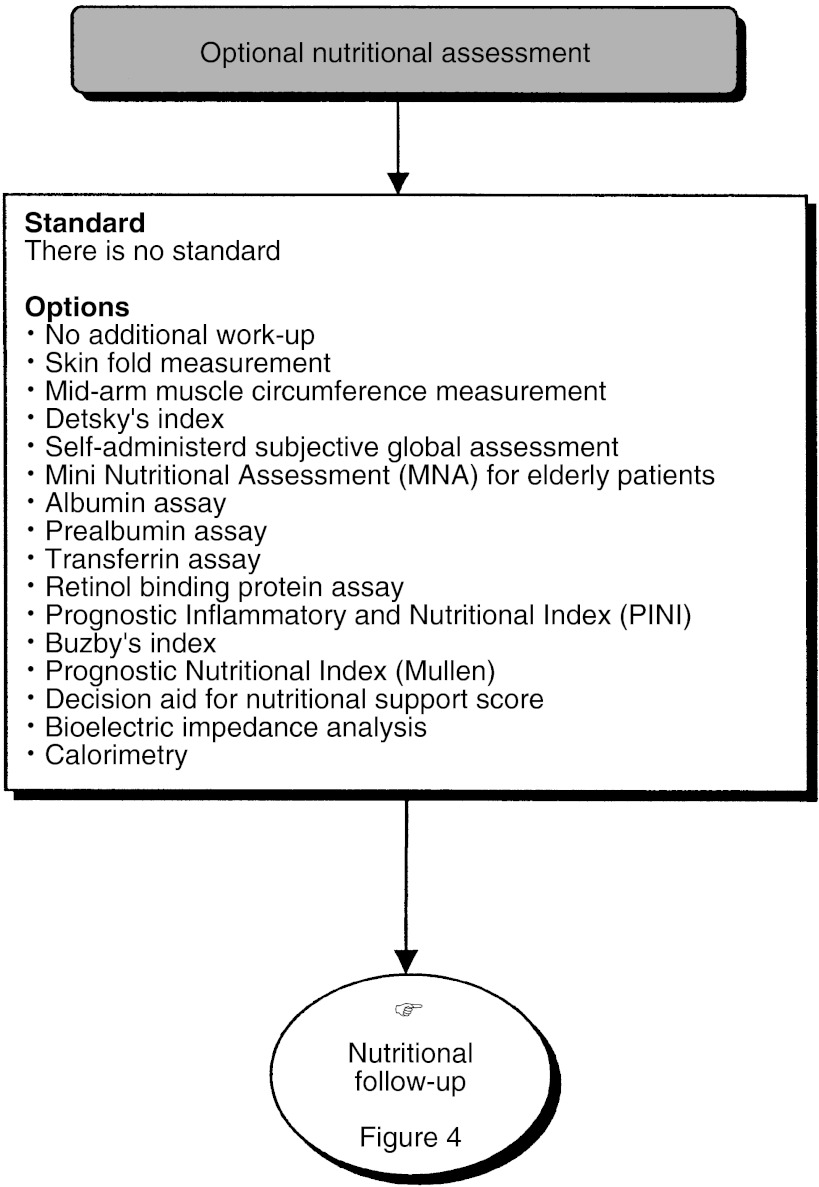
Optional nutritional assessment.

Additional nutritional assessment can be undertaken depending on the patient and their treatment. Detsky's subjective global assessment or Buzby's nutritional risk index should be used for patients undergoing major surgery (standard).

For patients who suffer from malnutrition, the assessment can be complemented by Detsky's subjective global assessment or Buzby's nutritional risk index (option).

In patients who suffer from malnutrition or who have undergone major surgery, the work-up can include an albumin and/or prealbumin assay and an assay for inflammatory proteins (orosomucoids, C-reactive protein) for the calculation, for example, of the prognostic inflammatory and nutritional index (option).

A mini nutritional assessment can be used in the work-up and follow-up of elderly patients (option).

## FOLLOW-UP OF NUTRITIONAL MANAGEMENT (FIGURE 4)

**Figure 4:**
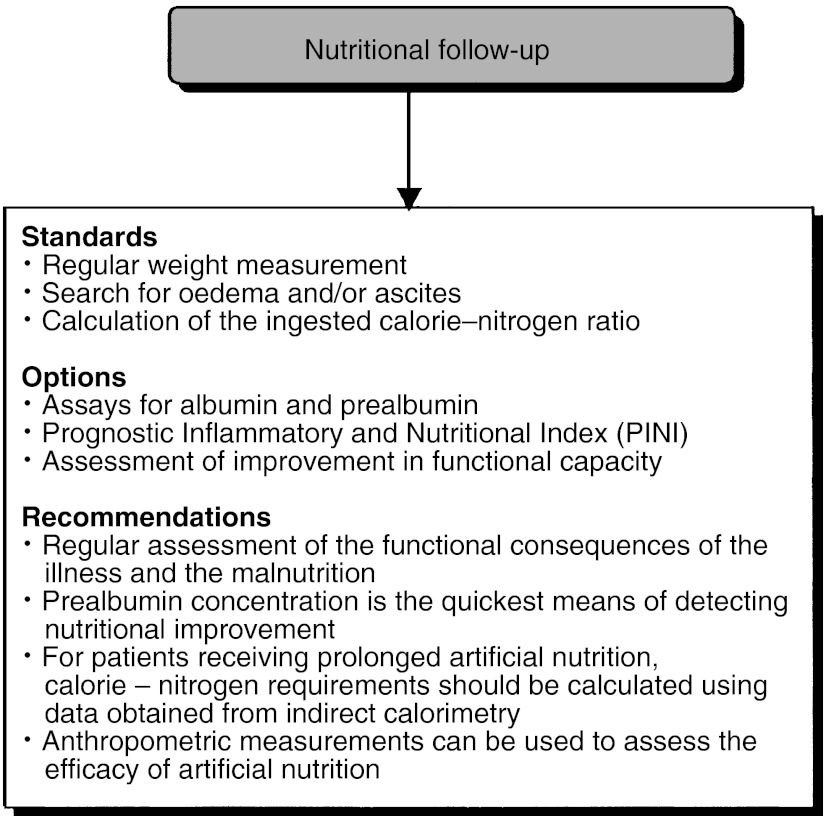
Nutritional follow-up.

Follow-up of the efficacy of the nutritional management should include regular weight measurement, plus an examination for oedema or ascites (standard). The calorie–nitrogen ratio intake should be determined regularly (standard). Follow-up of the efficacy of nutritional management can include the determination of serum albumin and prealbumin concentrations, a calculation of the prognostic inflammatory and nutritional index, and an assessment of any improvement in the patients' functional capacity and/or dynamometry (option). The patient's inflammatory state should be taken into consideration when interpreting albumin levels and the prognostic inflammatory and nutritional index (option). In patients undergoing prolonged artificial nutrition, the efficacy of the nutritional management can be assessed by measuring skin fold thickness and the mid-arm muscle circumference and by bioelectrical impedance analysis (options).

Regular assessment of the functional consequences of the cancer and any malnutrition is recommended. The concentration of prealbumin is the biological parameter that probably enables the quickest assessment of any nutritional improvement. In patients undergoing prolonged artificial nutrition, the calorie–nitrogen requirement should be calculated using data obtained from indirect calorimetry. Anthropometric measurements can be used to assess the efficacy of artificial nutrition.

## Appendix

### Reviewers

E Aden-Combes (CHRU, Pavillon Junod, Toulouse, France), F Arnaud-Battandier (Nestlé Clinical Nutrition, Sèvres, France), A Bataillard (FNCLCC, Paris, France), J Béal (Centre Oscar Lambret, Lille, France), B Besnard (Centre Paul Papin, Angers, France), C Bila (Institut Jules Bordet, Bruxelles, Belgium), P Bouletreau (Hôpital Hôtel Dieu, Lyon, France), A Bouvet (Centre François Baclesse, Caen, France), R Bugat (Institut Claudius Regaud, Toulouse, France), C Chambrier (Hôpital Hôtel Dieu, Lyon, France), R Benamouzig (Hôpital Avicenne, Bobigny, France), C Bonneteau (Institut Bergonié, Bordeaux, France), S Champetier Institut Gustave Roussy, Villejuif, France), M Claude (Institut Jean Godinot, Reims, France), D Combret (Hôpital Neurologique Cardiologique, Lyon, France), F Cometto (Centre Jean Perrin, Clermond-Ferrand, France), V Colomb (Hôpital Necker, Paris, France), F Dayot (Centre René Gauducheau, Nantes, France), JC Desport (CHU Dupuytren, Limoges, France), N Duval (Centre François Baclesse, Caen, France), AM Favreau (CHU, Angers, France), C Finck (Centre Paul Strauss, Strasbourg, France), A Freby-Lehner (Centre René Huguenin, Saint-Cloud, France), V Garabige (Institut Curie, Paris, France), C Garaud (Hôpital Clamart, France), MC Gouttebel (Centre hospitalier, Roanne, France), D Hoguet (Centre Oscar Lambret, Lille, France), M Kalsh (Institut Gustave Roussy, Villejuif, France), D Kere (Centre Val d'Aurelle Montpellier, France), F Lakdja (Institut Bergonié, Bordeaux, France), D Lefebvre (Centre Oscar Lambret, Lille, France), X Lefevre (Hôpital Michallon, Grenoble, France), D Lescut (CHR-Hôpital Claude Huriez, Lille, France), C Massoud (Centre François Baclesse, Caen, France), JC Melchior (CHU-Hôpital Bichat, Paris, France), J Meuric (Institut Curie, Paris, France), J Meynadier (Centre Oscar Lambret, Lille, France), F Montange (Centre Alexis Vautrin, Vandoeuvre Lès Nancy, Nancy, France), C Montane (Institut Claudius Regaud, Toulouse, France), G Nitenberg (Institut Gustave Roussy, Villejuif, France), T Oszustowicz (Centre Oscar Lambret, Lille, France), I Parmentier (CHRU, Lille, France), G Picard (Hôpital St Louis, Paris, France), B Poirée (Centre François Baclesse, Caen, France), P Pouillart (Institut Curie, Paris, France), MC Puissant (siège APHP, Paris, France), S Puel (Centre Val d'Aurelle, Montpellier, France), B Raynard (Hôpital Antoine Béclère, Clamart, France), C Rives (Institut Gustave Roussy, Villejuif, France), A Rivière (Centre François Baclesse, Caen, France), J Rodriguez (Institut Curie, Paris, France), N Rosiod (Hôpital Charles Foix, Ivry sur Seine, France), G Rossignol (Institut Gustave Roussy, Villejuif, France), P Roux-Bournay (Centre Léon Bérard, Lyon), B Saint-Aubert (Centre Val d'Aurelle, Montpellier), S Schneider (Hôpital de l'Archet, Nice, France), P Senesse (Centre Val d'Aurelle, Montpellier), M Simon (Centre Alexis Vautrin, Nancy, France), M Tran (Centre François Baclesse, Caen), J Vaccari (Hôpital Michallon, Grenoble, France), J Vedrenne (Institut Curie, Paris, France).
